# Is there a processing advantage for verb-noun collocations in Chinese reading? Evidence from eye movements during reading

**DOI:** 10.3389/fpsyg.2023.1235735

**Published:** 2023-08-30

**Authors:** Hui Li, Xiaolu Wang, Kevin B. Paterson, Hua Zhang, Degao Li

**Affiliations:** ^1^School of International Studies, NingboTech University, Ningbo, China; ^2^School of International Studies, Zhejiang University, Hangzhou, China; ^3^School of Humanities and Communication Arts, Western Sydney University, Sydney, NSW, Australia; ^4^School of Psychology and Vision Sciences, University of Leicester, Leicester, United Kingdom; ^5^College of Chinese Language and Literature, Qufu Normal University, Qufu, China

**Keywords:** eye movements, collocations, contextual predictability, collocation strength, Chinese reading

## Abstract

A growing number of studies show a processing advantage for collocations, which are commonly-used juxtapositions of words, such as “joint effort” or “shake hands,” suggesting that skilled readers are keenly perceptive to the occurrence of two words in phrases. With the current research, we report two experiments that used eye movement measures during sentence reading to explore the processing of four-character verb-noun collocations in Chinese, such as 修改文章 (“revise the article”). Experiment 1 compared the processing of these collocations relative to similar four-character expressions that are not collocations (e.g., 修改结尾, “revise the ending”) in neutral contexts and contexts in which the collocation was predictable from the preceding sentence context. Experiment 2 further examined the processing of these four-character collocations, by comparing eye movements for commonly-used “strong” collocations, such as 保护环境 (“protect the environment”), as compared to less commonly-used “weak” collocations, such as 保护自然 (“protect nature”), again in neutral contexts and contexts in which the collocations were highly predictable. The results reveal a processing advantage for both collocations relative to novel expressions, and for “strong” collocations relative to “weak” collocations, which was independent of effects of contextual predictability. We interpret these findings as providing further evidence that readers are highly sensitive to the frequency that words co-occur as a phrase in written language, and that a processing advantage for collocations occurs independently of contextual expectations.

## Introduction

Formulaic language (i.e., frequently occurring word sequences) is widely used in spoken language and written texts. Collocations are an example of formulaic language in which two or more words are habitually juxtaposed to create phrases, such as “black coffee,” “heavy rain” or “in a moment,” that are used regularly in spoken and written language (Biber et al., 1999; Vilkaite and Schmitt, 2019). The collocations are dissimilar to other word conjuctions, such as compound words (e.g., “headline,” “supermarket”), and hyphenated compounds (e.g., “baby-faced”), where words are combined to create a new or distinctive meaning. Collocations, by contrast, are simply commonly used sequences of words that are highly familiar to language users. According to one theoretical approach, the habitual utilization of collocations leads to their becoming lexicalized, which means they are perceived as a single language unit in the mind (e.g., Wray, 2002; Underwood et al., 2004; Conklin and Schmitt, 2008, 2012; Siyanova-Chanturia et al., 2011; Zang, 2019). However, an alternative to this view holds that the language processor computes statistics of word co-occurrences. It is argued to allow the language processor to exploit redundancy in the linguistic input, so that familiar or over-used sequences of co-occurring words can be processed more efficiently (e.g., McDonald and Shillcock, 2003a,b). This use of formulaic language is also considered a hallmark of linguistic proficiency, especially in the second-language learning literature where it is viewed as essential to the development of linguistic competence in non-native readers and speakers.

From a psycholinguistic perspective, there is considerable interest in understanding whether formulaic language is associated with specific processing advantages, as this may shed new light on how language is processed in the brain. One approach to this issue has involved comparing the processing of collocations relative to non-collocations. For instance, some researchers have used adaptations of the lexical decision task, in which participants must judge whether a linguistic stimulus is composed of real words or not (Ellis et al., 2009; Durrant and Doherty, 2010; Wolter and Yamashita, 2014). For example, Durrant and Doherty (2010) used a variant of this task in which pairs of words were presented sequentially and participants had to judge whether the second item in each pair was a real word or not. When the second item was a real word, there was a relationship between the two words, such that word pair formed either a collocation (e.g., “spoken word”) or a non-collocative phrase (e.g., “lower word”). This was shown to influence the time taken to judge that the second word in each pair (i.e., “word”) is a real word, with faster responses when it belonged to a collocation. Crucially, such findings suggest that words are recognized more quickly when they are part of a collocation than a non-collocative phrase.

In other research, eye movement measurements were utilized to determine if a comparable processing benefit is present when collocations and non-collocations are read naturally as part of a sentence. This methodology is based on the assumption that there is a close yoking between where a reader is looking and for how long and the cognitive processes involved in recognizing words during reading (e.g., Rayner, 1998, 2009; Liversedge and Findlay, 2000). In particular, numerous studies (for reviews, see Rayner, 1998, 2009) show that the time spent looking at (i.e., fixating) words during reading is sensitive to linguistic factors that affect its recognition, including a word’s frequency of usage in written language (with longer fixation times for words that are used less frequently) and its predictability from the prior linguistic context (with longer fixation times for words that are less predictable). Such findings have been crucial to the construction of computational reading models, including the highly influential E-Z Reader model (e.g., Reichle et al., 1998, 2003). A fundamental assumption of these computational models, such as the E-Z Reader model, is that information about word frequency rather than phrase frequency is computed (for discussion, see Cutter et al., 2014). Accordingly, research indicating that collocations are processed faster than non-collocative phrases is potentially important for the future development of such models, by demonstrating the need for theoretical models of the reading process to take account of the usage of phrases as well as words (for discussion, see Zang, 2019).

An example of such findings is from an eye-movement study demonstrating that collocations (e.g., “provide information”) are fixated for less time during reading as compared to non-collocative phrases (e.g., “compare information”) (Vilkaite, 2016). Other evidence comes from eye movement studies of the processing of collocations that vary in their frequency of usage. In these studies, two measures, namely, phrasal frequency (Gries and Ellis, 2015) and mutual information (MI; Hunston, 2002) are often computed. Phrasal frequency is a measure of how often words are used together in a phrase (within a language corpus), while MI involves computing how often the words are used together rather than separately. Sonbul (2015) examined eye movements for synonymous adjective-noun pairs that were either “strong” collocations, such as “fatal mistake,” which have high phrasal frequency and MI, “weaker” collocations, like “awful mistake,” which have lower phrasal frequency and MI, and phrases such as “extreme mistake,” which have very low phrasal frequency and MI. The findings showed that fixation times on these phrases was shortest for the “strong” collocations, longer for the “weak” collocations, and longest of all for the “very weak” collocations, revealing that eye movements are sensitive to the frequency of usage of these words as phrases.

An alternative approach adopted by McDonald and Shillcock (2003a,b) considered whether such effects might be characterized in terms of the language processing system tracking transitional probabilities, which can be defined as the probability of one word being followed by another in written language. Unlike phrasal frequency and MI, transitional probabilities do not rely on the proximity of words in a text. In an eye-tracking experiment, McDonald and Shillcock observed shorter fixation times for phrases with a high transitional probability like “accept defeat” relative to phrases with a lower transitional probability like “accept losses.” Based on these findings, McDonald and Shillcock argued that readers can utilize transitional probabilities to take advantage of the redundancy in language input, facilitating quicker processing of text. Frisson et al. (2005), however, argued that transitional probabilities could be part of contextual predictability, such that words with high transitional probabilities would be likely to co-occur in specific contexts. To test this hypothesis, they used similar verb-noun phrases to the ones McDonald and Shillcock employed in their experiments, and the phrases were presented in sentence contexts in which the phrases were either highly predictable or not. Comparing eye movements for the phrases, Frisson et al. found that contextual predictability, and not transitional probabilities, influenced fixation times for these phrases, and argued that transitional probability is not a separate statistical measure but part of contextual constraint on co-occurrence of words.

Accordingly, it is reasonable to assume that the effects of collocation frequency found in previous studies might represent contextual predictability on word co-occurrences. Thus, Li et al. (2021) followed Frisson et al.’s approach and used eye movement measures to explore the processing of collocations versus non-collocative phrases in predictive versus neutral contexts. Specifically, they compared the processing of “strong” adjective-noun collocations, such as “black coffee,” which had high MI scores and phrasal frequency, with “weak” collocations, such as “bitter coffee,” which had lower MI scores and phrasal frequency. The findings revealed that the effects of collocation strength were separate from the effects of contextual constraints on fixation times for the verb-noun phrases, suggesting that “strong” collocations are processed faster than “weak” collocations and the processing advantage is not attributable to contextual predictability. The experiment therefore provided evidence for readers’ sensitivity to the frequency of collocation use.

The present research extends these investigations to examine the processing of four-character collocations in Chinese. In written Chinese, a text is composed of box-like logograms called characters. Over 70% of commonly used Chinese words consist of two characters. At the same time, there is rich usage of four-character collocations (e.g., 保护环境, meaning “protect the environment”) created from the pairing of two two-character words (e.g., 保护, meaning “protect,” and 环境, meaning “environment”). These share some similarities to four-character Chinese idioms, which constitute 95% of Chinese idioms, and which are processed faster than novel phrases according to previous eye movement researches (e.g., Yu et al., 2016). However, collocations differ from idioms by being more semantically transparent and more widely used. We therefore attempt to investigate whether there are processing advantages for these collocation phrases.

Evidence from studies using a variant of the lexical decision task suggests that such a processing advantage exists. For example, Lv and Shi (2016) showed that reaction times for four-character verb-noun combinations were faster for collocations (e.g., 采取措施, “take measures”) as compared to non-collocative phrases with a similar meaning (e.g., 采用措施, “use measures”). More recently, Jiang et al. (2020) examined readers’ eye movements for four-character verb-noun collocations (e.g., 参加会议, “attend a meeting”) and non-collocative control phrases (e.g., 参加游戏, “attend a game”) that were embedded interchangeably in the same sentence frame. The results showed that readers had shorter fixation times for the collocations than the control phrases, suggesting a processing advantage for the collocations.

## Method

To further investigate this issue, the present study has conducted two experiments. In Experiment 1, we examined eye movements for four-character collocations and control phrases in neutral contexts and contexts in which the collocation was highly predictable. In Experiment 2, we compared the processing of “strong” collocations (i.e., with high phrasal frequency and MI) against “weak” collocations (i.e., with lower phrasal frequency and MI) to establish whether a processing advantage for collocations would be modulated by contextual constraints or observed independently of effects of context.

### Experiment 1

Experiment 1 used eye movement measures to compare the processing of four-character verb-noun collocations, such as 修改文章 (“revise the article”) and four-character non-collocative control phrase with the same verb but a different noun, such as 修改结尾 (“revise the ending”). We aimed to establish whether a collocation processing advantage would be observed in fixation times for these phrases during reading, and whether such effects are modulated by context or observed independently of context. In making these comparisons, we draw a distinction between the measures of eye movements which show sensitivity to first-pass processing (i.e., the processing which occurs at the beginning) of a phrase and those sensitive to the later processing of the phrase (e.g., Rayner, 1998, 2009). This difference is important as it allows us to draw inferences about the time course of any observed effects and, specifically, whether effects are observed during the early processing of phrase, during which words will be recognized, or during some later stage of processing that might reflect how easily the phrase can be integrated with context. In addition, following Li et al. (2021), we report two sets of analyses. One focused on the processing of the phrase as a whole and the other focused on the more localized processing of the noun in the four-character verb-noun phrase. An advantage of this second analysis is in revealing whether collocative status and contextual constraints can guide the recognition of this critical word. A further advantage is that this approach allows us to conduct comparisons of these effects across an identical linguistic constituent in the collocation and control phrases (noting that while we have strived to match carefully the different verbs used in collocation and control phrases in terms of critical features, these nevertheless are different words).

### Participants

Forty-four Chinese native speakers (30 female) aged 18–22 years (*M* = 19.8 years, SD = 1.1) from Zhejiang University were paid to participate in the experiment. All reported having normal or corrected-to-normal vision. All participants reported being right-handed and having no history of neurological, psychiatric, or reading impairment.

### Stimuli and design

The stimuli were 34 pairs of four-character verb-noun phrases, each comprising two two-character words. These were obtained from a database of 3,783 commonly-used two-character words (Song and Li, 2021). Each pair included a collocation (e.g., 修改文章, “revise the article”) and a control phrase with the same verb but a different noun (e.g., 修改结尾, “revise the ending”). The nouns in the collocations and control phrases were matched for lexical frequency using the SUBTLEX-CH-CHR database (Cai and Brysbaert, 2010), and for visual complexity in terms of their number of strokes. The collocative strength of each phrase (i.e., the extent to which the verb and noun combinations are used together habitually) was calculated using both phrasal frequency and Mutual Information (MI) scores. Phrasal frequency indicates how often a noun and verb combination co-occurs as a phrase (Gries and Ellis, 2015). MI provides a log-normalized measure, a ratio of the frequency of a word co-occurring in a phrase relative to the frequency of separate use (Hunston, 2002). Phrasal frequency scores for collocation and control phrases were obtained from the BCC corpus (Gou et al., 2015). MI scores were computed based on the formula MI = log_2_ $\frac{O}{E}$ (Durrant and Doherty, 2010), where *O* and *E* were the observed and expected occurrences of each phrase in the BCC corpus. As shown in Table 1, the collocation and control phrases differed significantly in phrasal frequency and MI.

**Table 1:** Stimulus characteristics for Experiment 1.

Characteristic	Collocation	Control phrase	*t*	*p*
Phrasal Frequency	703.8 (213.2)	0.4 (0.2)	3.299	< 0.05
MI	9.7 (0.4)	4 (0.2)	20.359	< 0.001
Noun Frequency	24.4 (4.1)	23.4 (4.0)	0.184	0.855
No. of Strokes	14.6 (0.7)	14.5 (0.7)	0.089	0.93

The Standard Error of the Mean is shown in parentheses.

Each collocation and control phrase pair was embedded interchangeably in two sentence frames. The sentences were 15 to 21 characters long ( *M* = 19.5 characters, *SD* = 2.2 characters). The collocation or control phrase always appeared near the middle of the sentence which was presented as a single line of text. One sentence frame provided a context in which the collocation was predictable, while the other sentence frame provided a neutral context in which the collocation was not predicted but also not anomalous. An example set of sentence frames and collocation/control phrase pair is shown in Table 2.

**Table 2:** An example stimulus in Experiment 1.

Context	Phrase type	Sample sentence
Predictable	Collocation	该俱乐部只为会员**提供服务**并不对外开放。 This club only **provides services** for the members and is not open to the public.
	Control	该俱乐部只为会员**提供号码**并不对外剬布。 This club only **provides numbers** for the members and does not disclose them to the public.
Neutral	Collocation	这个地方只为李娜**提供服务**并不对外开放。 This place only **provides services** for Li Na and is not open to the public.
	Control	这个地方只为李娜**提供号码**并不对外剬布。 This place only **provides numbers** for Li Na and does not disclose them to the public.

Contextual predictability was assessed using a modified cloze procedure. In this, 20 participants (young adults from the same population as participants in the experiments) were asked to write continuations for sentence fragments which were truncated prior to the target phrase (i.e., the collocation / control phrase). If the continuation included the target phrase or a word related to its verb, the collocation was regarded as predictable. For example, if the target collocation was 提供服务 (“provide services”), continuations related to the concept of 提供 (i.e., “provide”) were taken to demonstrate that the phrase was predictable from the prior sentence context. A collocation was considered predictable if more than 50% of continuations met this criterion, and less predictable if fewer than 20% of continuations met this criterion. Following this procedure, we selected sentences and collocation combinations in which the collocation was significantly more predictable in the predictable frame (*M* = 71.1%, SD = 2.5%) than the neutral frame (*M* = 0.9%, SD = 0.5%; *t*_(66)_ = 27.182, *p* < 0.001). Another 20 young adults assessed the naturalness of predictive and neutral sentences which included either the collocation or the control phrase, using a 5-point Likert scale (where 1 = not natural and 5 = very natural). Sentence naturalness did not differ significantly as a function of sentence type (predictable context, *M* = 4.2, SD = 0.5, neutral context, *M* = 4.0, SD = 0.4, *F* (1, 19) = 1.14, *p* = 0.299), phrase (collocation, *M* = 4.0, SD = 0.4, control phrase, *M* = 4.0, SD = 0.3, *F* (1, 19) = 0.909, *p* = 0.352), or an interaction of these factors (*F*(1, 19) = 1.060, *p* = 0.316), indicating that the collocation and control phrases were similarly acceptable in the predictive and neutral sentence frames.

The sentence and phrase combinations were pseudo-randomly allocated to two presentation lists. For one list, half the phrase pairs were presented with the collocation in a predictive sentence context and the control phrase in a neutral sentence context, and the other half were presented with the collocation in a neutral sentence context and the control phrase in a predictive sentence context. This allocation of collocations and control phrases to predictive and neutral contexts was reversed for the other list. Participants were pseudo-randomly allocated to a list so that an equal number of participants viewed each list. This assured that, while each collocation and control phrase was viewed once by each participant and in only one sentence context, each phrase was read equally often in a predictive or neutral sentence context across the experiment.

Each list contained 72 sentences with 17 sentences in each treatment level of context type by phrase type. Intermingled with these, each list included additional 72 filler sentences that were similar to the critical sentences in length and readability. Each list also began with 8 practice sentences. The experiment had a 2 (phrase type: collocation or control) x 2 (context type: predictable or neutral) within-participants design.

### Apparatus and procedure

During binocular reading, the gaze location of each participant’ right eye was recorded every millisecond using an EyeLink 1,000 Plus tower-mounted eye tracker. Sentences were displayed on a 19-inch monitor (1,024 × 768 pixels) in Song font as black text on a light grey background. At approximately 71 cm viewing distance, each character subtended approximately 0.8° of visual angle.

Participants took part individually. On arrival, each participant had the experimental procedure explained to them and was asked to read the sentences for comprehension. The participant was then seated at the eye-tracker with the head on a chin and forehead rest to reduce any potential movements of the head. A three-point horizontal calibration was utilized to ensure spatial accuracy of at least 0.35° across the same line as sentence stimuli would be presented. To maintain this resolution, we checked the accuracy of calibration before we presented each trial and recalibrated if necessary. Each trial started with a presentation of a fixation square which is the same size as a character. Once the participant fixated this square, the square vanished and the initial character took its place in the sentence. Once finishing reading, the participant pressed a response button and the sentence disappeared, replaced by a new trial or a yes/no comprehension question on 25% of trials. Participants answered the comprehension question by pressing one of two response buttons, and these responses were recorded by the computer. The experiment lasted approximately 30 min for each participant.

### Data analysis

Linear mixed effects models (LMEM, Baayen et al., 2008) and the lme4 package (version 1.1–26; Bates et al., 2012) in the R environment (R Core Team, 2019) were used to analyze the remaining data. We designated participants and stimuli as crossed random effects for all analyses, whereas context type and sentence type were designated as fixed factors. A model with a completely random structure was employed (Barr et al., 2013). If the entire model failed to converge, its randomized structure was adjusted by progressively removing correlations between factors and then interactions, until convergence was achieved. The “contr.sdif” function in the MASS package was utilized to specify the comparisons of fixed factor levels (Venables and Ripley, 2002). Simple effects abakyses were performed using the emmeans package (Lenth, 2017). For all analyses, *t* > 1.96 was considered statistically significant.

Separate analyses were conducted for the target phrase as a whole and for its noun (see Carrol and Conklin, 2014; Vilkaite, 2016; Li et al., 2022). For full phrase analyses, we report a measure of the initial (i.e., first-pass) processing of the phrase; namely first-pass reading time (FPRT, the total number of fixations from the phrase’s initial forward-directed fixation until a progressive eye movement to its right or a regression to its left), alongwith measures that can detect changes in how the phrase is processed at a later time, namely regression-path reading time (RPRT, the total duration of fixation from the initial fixation on a phrase until the eye moves progressively to the right, encompassing any fixations that occur after a regression from that phrase; Liversedge et al., 1998), regressions-in (the likelihood of reverting back to the original phrase through regression), and total reading time (TRT, the total number of fixations on the phrase regardless of when these are made during sentence processing). The probabilities of readers either skipping the target phrases or making a regression from these phrases during first-pass reading were low and so are not included in analyses of first-passing processing.

For the noun analyses, we examined measures of the first-pass processing; namely, word-skipping (the likelihood of not fixating the noun in initial processing), first-fixation duration (FFD, the duration of the initial progressive fixation on the noun), single-fixation duration (SFD, the duration of the initial progressive fixation on nouns receiving solely one fixation without any other fixations), regressions-out, and gaze duration (the total number of initial fixations on the noun, equivalent to first-pass reading time for a single word region). Additionally, we examined measures sensitive to the later processing of the noun; namely regression-path reading time, and total reading time.^1^

## Results

Accuracy answering comprehension questions averaged 93% (> 80% for all participants), indicating that the participants had understood the sentences. Prior to data analysis, and following standard procedures, adjacent fixations were combined and short fixations (less than 80 ms) and long fixations (more than 1,000 ms) were deleted, affecting 3.7% of the data. In addition, fixations more than 2.5 *SD*s from the mean per condition for each participant were removed as outliers (affecting 2.2% of fixations).

### Phrase analysis

Descriptive and inferential statistics for the critical phrases are summarized in Tables 3, 4, respectively. A main effect of phrase type was obtained in all the measures. Reading times (in FPRT, RPRT and TRT) were shorter for the collocations than for the control phrases. A main effect of context type was significant in all measures. Reading times were shorter for phrases in predictable than neutral contexts. The interaction was not significant between the influences of collocation and context type in any of the measures.

**Table 3:** Mean eye movements for the collocation / control phrase in Experiment 1.

	Predictive context		Neutral context	
	Collocation	Control	Collocation	Control
FPRT (ms)	395 (9)	446 (10)	414 (9)	456 (10)
RPRT (ms)	520 (11)	689 (17)	580 (13)	736 (17)
TRT (ms)	520 (11)	689 (17)	580 (13)	736 (17)

The Standard Error of the Mean is shown in parentheses. FPRT, first-pass reading time, RPRT, regression-path reading time, TRT, total reading time.

**Table 4:** Summary of statistical effects for the collocation / control phrases in Experiment 1.

Factor	Statistic	FPRT	RPRT	TRT
Intercept	β	425.74	628.46	628.46
	SE	20.19	36.66	36.66
	t/z	21.09	17.14	17.14
Collocation (collocation – control)	β	46.16	162.12	162.12
	SE	7.86	11.92	11.92
	t/z	5.88^*^	13.60^*^	13.60^*^
Context (neutral - predictable)	β	−14.02	−53.32	−53.32
	SE	7.86	11.92	11.92
	t/z	−1.79	−4.47^*^	−4.47^*^
Context x Collocation	β	10.04	13.70	13.70
	SE	15.71	23.85	23.85
	t/z	0.64	0.58	0.58

Asterisks indicate statistically significant effects, *p* < 0.05. FPRT, first-pass reading time, RPRT, regression-path reading time, TRT, total reading time.

### Noun analysis

Descriptive and inferential statistics for the critical noun were summarized in Tables 5, 6, respectively. In all measures, it was found that there were main effects of phrase type. Compared to the control nouns, nouns in collocations had higher skipping rates, produced fewer first-pass regressions from the phrase, and had shorter first-pass reading times (i.e., FFD, SFD, and GD) and reading times sensitive to later processing (i.e., RPRT and TRT). A main effect of context type was obtained in regressions-out and in reading time measures sensitive to later processing associated with contextual integration (i.e., RPRT, TRT). As with the phrase analysis, there was no interaction between context and phrase type.

**Table 5:** Means for the critical noun in Experiment 1.

	Predictable context		Neutral context	
	Collocation	Control	Collocation	Control
SKIP (%)	22 (2)	14 (1)	19 (1)	13 (1)
FFD (ms)	243 (3)	251 (4)	244 (3)	254 (3)
SFD (ms)	245 (4)	256 (5)	248 (5)	256 (5)
GD (ms)	263 (5)	276 (5)	256 (4)	274 (5)
RO (%)	10 (1)	20 (2)	14 (1)	23 (2)
RPRT (ms)	376 (10)	510 (16)	423 (12)	553 (15)
TRT (ms)	312 (7)	388 (9)	338 (8)	400 (9)

The Standard Error of the Mean is shown in parentheses. SKIP = word-skipping, FFD, first-fixation duration, SFD, single-fixation duration, GD, gaze duration RO, Regressions-out, RPRT, regression-path reading time, TRT, total reading time.

**Table 6:** A summary of statistical effects for the noun region in Experiment 1.

Factor	Statistic	SKIP	FFD	SFD	RO	GD	RPRT	TRT
Intercept	β	−1.82	246.31	252.08	−1.89	264.52	452.07	351.13
	*SE*	0.14	5.39	6.20	0.15	7.04	26.45	15.61
	*t/z*	−13.26	45.73	40.69	−12.78	37.59	17.09	22.49
Collocation (collocation–control)	β	−0.57	9.72	12.73	0.77	16.67	136.15	72.96
	*SE*	0.10	3.53	4.23	0.12	4.27	11.85	7.35
	*t/z*	−5.59^*^	2.75^*^	3.01^*^	6.64^*^	3.90^*^	11.49^*^	9.92^*^
Context (neutral - predictable)	β	0.13	−2.40	−1.95	−0.31	3.76	−45.59	−18.69
	*SE*	0.10	3.18	4.21	0.12	5.08	11.85	7.35
	*t/z*	1.24	−0.76	−0.46	−2.71^*^	0.74	−3.85^*^	−2.54^*^
Context x Collocation	*β*	−0.11	−0.50	5.37	0.20	−2.27	12.99	19.24
	*SE*	0.21	6.34	8.43	0.23	8.55	23.71	14.71
	*t/z*	−0.53	0.08	0.64	0.85	−0.27	0.55	1.31

Asterisks indicate statistically significant effects, *p* < 0.05. SKIP, word-skipping, FFD, first-fixation duration, SFD, single-fixation duration, GD, gaze duration RO, Regressions-out, RPRT, regression-path reading time, TRT, total reading time.

## Discussion

Our results revealed clear effects of both phrase type and contextual predictability for the whole phrases and for the noun in each phrase in both first-pass and later eye movement measures, with no indication of an interaction effect. The effect of phrase type was due to shorter reading times for collocations compared to non-collocative phrases, and for the noun in the collocations relative to the same noun in the control phrase. Our finding that collocations were read faster than non-collocations provides evidence for a collocation processing advantage, consistent with Jiang et al.’s (2020) findings for four-character phrases. Moreover, the finding that these effects emerged in first-pass processing, including of the noun in the phrase, is consistent with collocative phrases being recognized more quickly than non-collocative phrases. These findings therefore provide evidence that readers use the co-occurrence information provided by collocations to predict upcoming words, so that these words are recognized more easily, in line with usage based accounts (e.g.,. Bybee, 2006).

The effects of contextual predictability we obtained confirmed the effectiveness of our manipulation of contextual constraints. Reading times for the collocation and control phrases (and their nouns) were faster in predictive than neutral contexts, consistent with effects of word predictability in reading (Rayner and Well, 1996; see Rayner, 2009). These effects emerged relatively late in the eye movement record; however, appearing first in regression-path reading times for the full phrase and in regressions-out for the nouns, indicating that contextual constraint served to aid the integration of the four-character words with the context. Specifically, readers were less likely to look back in the sentence and engage in re-reading of the prior sentence context when the phrases were contextually predictable.

Finally, the absence of an interaction between phrase type and contextual predictability was consistent with previous findings by Li et al. (2021) indicating that the processing advantage for collocations is observed independently of contextual status, and so does not appear to represent a specific type of contextual predictability (see Frisson et al., 2005).

Experiment 1 provides evidence for a processing advantage for four-character verb-noun collocations relative to matched non-collocative phrases that was independent of contextual predictability. To further investigate this processing advantage, Experiment 2 investigated whether readers could detect the differences in collocation frequency, by analyzing how “strong” and “weak” collocations were processed in neutral and predictive contexts.

### Experiment 2

Experiment 1 confirmed that four-character collocations are processed faster than control phrases in both predictive and neutral sentence contexts. Experiment 2 explored this processing advantage for four-character collocations further, by examining whether readers are sensitive to co-occurrence frequencies. Specifically, we examined whether readers might also exhibit a processing advantage for “strong” four-character collocations, which have a high frequency of usage, relative to “weak” four-character collocations that are used less frequently. As in Experiment 1, these collocations were presented to readers in both predictive and neutral sentence contexts to establish whether any processing advantage is modulated by contextual predictability.

### Participants

Thirty-six participants (32 female; aged 18–23 years; *M* = 20.6 years, SD = 1.2) were native Chinese speakers recruited from Zhejiang University. None had participated in Experiment 1. All participants were paid to take part in this experiment. All reported being right-handed, with normal or corrected vision and no history of neurological, psychiatric, or reading impairment.

### Stimuli and design

Stimuli consisted of 36 pairs of four-character verb-noun collocations where the members of each pair used the same verb (e.g., 保护, “protect”) but a different noun. Based on phrasal frequency and MI, one of each pair was categorized as a “strong” collocation (e.g., 保护环境, “protect the environment”) and the other as a “weak” collocation (e.g., 保护自然, “protect the nature”).

Characteristics of the strong and weak collocations are shown in Table 6. Following similar procedures to Experiment 1, the nouns in the strong and weak collocations were matched for lexical frequency using the SUBTLEX-CH-CHR database (Cai and Brysbaert, 2010), and for visual complexity in terms of their number of strokes. Phrasal frequency and MI were calculated using the BCC corpus (Gou et al., 2015). As shown in Table 7, strong collocations had significantly higher phrasal frequency and MI than the weak collocations.

**Table 7:** Stimulus characteristics of the strong and weak collocations used in Experiment 2.

Characteristic	Strong	Weak	*t*	*p*
Phrasal Frequency	837.42 (192.10)	222.72 (55.07)	3.076	< 0.05
MI	9.74 (0.40)	8.03 (0.39)	3.072	< 0.05
Noun Frequency	26.01 (3.93)	20.61 (2.84)	1.115	0.269
No. of Strokes	14.89 (0.78)	14.42 (0.56)	0.494	0.623

The Standard Error of the Mean is shown in parentheses.

Similarly to Experiment 1, each collocation pair was embedded interchangeably in two sentence frames. The sentences, including the target phrase, were 15 to 22 characters long (*M* = 19.7 characters, *SD* = 2.3 characters). One sentence frame was designed to provide a context in which the collocation was predictable, while the other sentence frame provided a neutral context in which the collocation was not predictable but also not anomalous. An example set of sentence frames and collocation/control phrase pair is shown in Table 2.

Contextual predictability was assessed using the same procedure as Experiment 1. The sentences were truncated prior to the collocation and 20 participants from the same population as the experiment participants provided a written continuation for each fragment. Predictability was assessed in terms of whether continuations included the collocations, its verb or words associated to its verb. For example, if the target collocation was 保护环境 “protect the environment,” then this phrase, the verb or continuations related to the verb were taken to demonstrate predictability. Collocations for which more than 50% of continuations met this criterion were categorized as predictable, whereas those for which fewer than 20% of continuations met this criterion were considered to be unpredictable. Following this procedure, we selected sentence and collocation combinations in which collocations were significantly more predictable in the predictable frame (*M* = 63.4%, SD = 2.3%) than the neutral frame (*M* = 0.2%, SD = 0.2%; *t*_(66)_ = 27.641, *p* < 0.001). Another 20 students assessed the naturalness of a sentence using a Likert scale consisting of five points, where 1 indicated “not natural” and 5 indicated “very natural.” Naturalness scores were high in both predictable contexts (strong collocations, *M* = 4.3, SD = 0.4, weak collocations, *M* = 4.2, SD = 0.5) and neutral contexts (strong collocations, *M* = 4.1, SD = 0.4, weak collocations, *M* = 4.1, SD = 0.4) and did not differ significantly as a function of context (*F* (1, 19) = 1.341, *p* = 0.261), collocation strength (*F* (1, 19) = 1.235, *p* = 0.280) or an interaction of these factors (*F* (1, 19) = 0.438, *p* = 0.516). This suggested that strong and weak collocations were acceptable within both sentence frames.

The sentences were pseudo-randomly divided into two lists. For one list, half the collocation pairs were shown with the strong collocation paired with the predictive context and the weak collocation paired with the neutral context, and the other half of the sentence contexts had a reverse allocation of strong and weak collocations. For the other list, this allocation of collocations pairs to sentence contexts was reversed. Each list therefore included 72 experimental trials, with 18 strong and 18 weak collocations in a predictive sentence frame and 18 strong and 18 weak collocations in a neutral sentence frame. An additional 72 filler sentences were added to each list. As in Experiment 1, the filler item were of similar length and readability as the experimental sentences.

Participants were pseudo-randomly allocated so that an equal number were assigned to each list. This ensured that each participant viewed the strong and weak collocation of each collocation pair only once and in a different context, viewed an equal number of strong and weak collocations in each context, and that each collocation was viewed an equal number of times in each context across the experiment. The experiment had a 2 (collocation type: stronger or weak) by 2 (context type: predictable or neutral) within-participants design.

### Apparatus and procedure

The same EyeLink 1,000 Plus (SR Research inc.) tower-mounted eye-tracker from Experiment 1was used to record gaze location from each participant’s right eye every millisecond during binocular reading. Sentences were displayed on a 19-inch monitor (1,024 × 768 pixels) using Song font as black text on a light grey background. At 71 cm viewing distance, each character subtended about 0.8° of visual angle.

As in Experiment 1, participants were instructed to read normally and for comprehension. They were then seated at the eye-tracker and a chin and forehead rest was used to minimize head movements. The eye-tracker was calibrated to each participant’s eye movements using a three-point horizontal calibration procedure (ensuring spatial error of less than 0.35°). Calibration accuracy was checked prior to each trial and the eye-tracker recalibrated as necessary to maintain high spatial accuracy. At the start of each trial, a fixation square appeared on the left side of the screen. Once this was fixated, the square disappeared and the sentence was presented with the first letter in the sentence replacing the square. Once the participant finished reading, he or she pressed a response button and the sentence would disappear to make way for a new trial or a comprehension question. On 25% of the trials, the participant was asked a comprehension question that required a yes or no answer. They responded by pressing one of two response buttons, and their answers were recorded by a computer. Each participant was involved in the experiment for approximately half an hour.

### Data analysis

Separate analyses were conducted for the target phrase as a whole and for its noun (see Carrol and Conklin, 2014; Vilkaite, 2016; Li et al., 2021). For the full phrase analyses, we report first-pass reading time (FPRT) as a measure of first-pass reading time, and regression-path reading time (RPRT) and total reading time (TRT) as measures of later processing. For the noun analyses, we report word-skipping (SKIP), first-fixation duration (FFD), single-fixation duration (SFD), and gaze duration (GD) as measures of first-pass reading. Additionally, we report regression-path reading time (RPRT) and total reading time (TRT), and as measures of later processing.^2^

## Results

The comprehension accuracy was a mean of 95%, with all participants scoring more than 80% correct. The eye movement data was screened in the same way as in Experiment 1. Following the standard procedure, adjacent fixations were combined and short fixations (less than 80 ms) and long fixations (more than 1,200 ms) were deleted (affecting 5% of the data). In addition, any fixations that were more than 2.5 standard deviations away from the mean for each participant in each condition were excluded as outliers, which impacted 1.5% of all the fixations.

### Phrase analyses

Descriptive and inferential statistics for the full phrase analyses are shown in Tables 8, 9, respectively. An effect of collocation strength was significant in all eye movement measures. This showed that strong collocations were processed more quickly compared to weak collocations, with this effect seen most clearly in late measures of processing. A main effect of context type was also significant in all eye movement measures. This showed that the four-character phrases were processed more quickly in predictive than neutral contexts, with these effects emerging during first-pass processing of the phrase and also observed in later measures. Crucially, there were no significant interactions between collocation strength and contextual predictability, suggesting that effects of collocation strength occurred independently of contextual constraint.

**Table 8:** Eye movement measures for the collocation phrases of Experiment 2.

	Predictable context		Neutral context	
	Strong collocation	Weak collocation	Strong collocation	Weak collocation
FPRT (ms)	345 (8)	344 (8)	369 (8)	397 (9)
RPRT (ms)	486 (12)	533 (14)	549 (13)	608 (14)
TRT (ms)	486 (12)	533 (14)	549 (13)	608 (14)

The Standard Error of the Mean is shown in parentheses. FPRT, first-pass reading time, RPRT, regression-path reading time, TRT, total reading time.

**Table 9:** Summary statistics for the collocation phrase of Experiment 2.

Factor	Statistic	FPRT	RPRT	TRT
Intercept	β	362.11	542.13	542.13
	SE	17.15	34.46	34.46
	t/z	21.11	15.73	15.73
Collocation (strong – weak)	β	14.45	55.09	55.09
	β	6.81	10.65	10.65
	SE	2.12^*^	5.17^*^	5.17^*^
Context (neutral - predictable)	β	−40.08	−71.52	−71.52
	β	9.73	18.30	18.30
	SE	−4.12^*^	−3.91^*^	−3.91^*^
Collocation x Context	β	−22.22	−4.79	−4.79
	β	13.62	21.31	21.31
	SE	−1.63	−0.23	−0.23

Asterisks indicate statistically significant effects, *p* < 0.05. FPRT, first-pass reading time, RPRT, regression-path reading time, TRT, total reading time.

### Noun analyses

Descriptive and inferential statistics for the collocation nouns are shown in Tables 10, 11, respectively. An effect of collocation strength was significant in word-skipping, gaze duration and in later reading time measures (i.e., RPRT and TRT). Compared to the weak collocations, the strong collocations had higher skipping rates, and shorter gaze durations during first-pass processing, and shorter regression-path and total reading times during later processing. A main effect of context type was also significant in word-skipping and single-fixation durations. This was due to higher word-skipping and shorter first-fixation durations for the noun in predictable compared to neutral contexts. No significant interactions were observed in any of the measures, again suggesting that effects of contextual strength were observed independently of contextual constraint.

**Table 10:** Eye movement measures for the collocation noun of Experiment 2.

	Predictable context		Neutral context	
	Strong collocation	Weak collocation	Strong collocation	Weak collocation
SKIP (%)	24 (2)	20 (2)	18 (2)	14 (1)
FFD (ms)	225 (3)	229 (3)	230 (3)	232 (3)
SFD (ms)	221 (4)	227 (4)	230 (5)	234 (5)
GD (ms)	244 (5)	254 (5)	244 (4)	255 (5)
RPRT (ms)	316 (8)	350 (10)	322 (8)	360 (9)
TRT (ms)	316 (8)	350 (10)	322 (8)	360 (9)

The Standard Error of the Mean is shown in parentheses. SKIP, word-skipping rate, FFD, first-fixation duration, SFD, single-fixation duration, GD, gaze duration, RPRT, regression-path reading time, TRT, total reading time.

**Table 11:** Summary statistics for the collocation noun of Experiment 2.

Factor	Statistic	SKIP	FFD	SFD	GD	RPRT	TRT
Intercept	β	−1.64	227.98	228.84	246.79	328.53	328.53
	*SE*	0.14	4.62	4.66	5.99	15.60	15.60
	*t/z*	−11.39	49.31	49.10	41.21	21.06	21.06
Collocation (strong – weak)	β	−0.27	3.10	4.96	10.48	36.03	36.03
	*SE*	0.10	3.07	4.16	4.29	7.78	7.78
	*t/z*	2.58^*^	1.01	1.19	2.45^*^	4.63^*^	4.63^*^
Context (neutral - predictable)	β	0.44	−5.45	−8.87	−2.34	−12.05	−12.05
	*SE*	0.13	3.11	4.17	6.15	11.41	11.41
	*t/z*	3.47^*^	−1.76	−2.13^*^	−0.38	−1.06	−1.06
Context x Collocation	*β*	0.02	4.01	4.17	1.26	−1.12	−1.12
	*SE*	0.21	6.14	8.34	8.57	15.57	15.57
	*t/z*	0.09	0.65	0.50	0.15	−0.08	−0.08

Asterisks indicate statistically significant effects, *p* < 0.05. SKIP, word-skipping, FFD, first-fixation duration, SFD, single-fixation duration, GD, gaze duration, RPRT, regression-path reading time, TRT, total reading time.

## Discussion

The results of Experiment 2 produced clear effects of collocation strength and contextual predictability. The effect of collocation strength was due to “strong” collocations that have a high frequency of written usage being read faster than “weak” collocations that are used less often in written language. The indication, therefore, is that readers can perceive the frequency of collocation use, so that collocative phrases that are encountered more often can be processed more easily. There was also some indication from analyses of eye movements for the noun region that this processing benefit may be a consequence of readers predicting the sequence of words in the “strong” four-character collocations, so that the noun was more likely to be skipped in “strong” compared to “weak” collocations.

As in Experiment 1, we also obtained an effect of contextual predictability, such that the “strong” and “weak” collocations were read faster in predictable than neutral contexts. This demonstrates the effectiveness of our manipulation of the phrases predictability. Consistent with Experiment 1 and previous research by Li et al. (2021), there was no interaction effect, and so no indication that contextual predictability modulated the collocation effect we observed. The processing advantage for “strong” collocations over “weak” collocations may reflect the readers’ knowledge of the frequency that words tend to co-occur in phrases rather than a specific form of contextual predictability.

### General discussion

With the present experiments, we investigated whether there is a processing advantage for collocations, which are habitual juxtapositions of two or more words that express a specific meaning, in Chinese reading. We focused on four-character verb-noun collocations, which have a rich usage in Chinese language. Following Li et al. (2021), we examined in Experiment 1 whether there is a processing advantage for these collocations relative to control phrases presented in neutral sentence contexts or sentences in which the collocation is predictable from the prior context. The results showed very clearly that the collocations were read faster than the control phrases in both neutral and predictive contexts, with no modulating effect of contextual predictability.

Experiment 2 was designed as a follow-up study to investigate whether readers’ eye movements are sensitive to variation in the frequency of collocation use, by comparing eye movements for “strong” collocations, which have a high frequency of usage, and “weak” collocations, which have a lower frequency of usage, again in neutral contexts and contexts in which the collocation is highly predictable. The results showed a processing advantage for “strong” collocations, with shorter fixation duration for the “strong” collocations relative to the “weak collocations.” The finding that skipping rates were higher for the noun in “strong” versus “weak” collocations additionally suggested that the word sequence was more predictable for collocations which are frequently used.

Our findings were in line with other research showing a specific processing advantage for four-character collocations in Chinese reading (Lv and Shi, 2016; Jiang et al., 2020). The findings are also consistent with the evidence from other research on eye movements showing a processing advantage for collocations over non-collocations (Vilkaite, 2016), and for “strong” collocations with a high frequency of usage over “weaker” collocations with a lower frequency of usage (Sonbul, 2015). Finally, the present findings are consistent with Li et al.’s (2021) finding that a collocation processing advantage is observed regardless of whether phrases are read in a neutral sentence context or one where the collocation is contextually predicted. This latter finding is important in the context of the claim that co-occurrence statistics (specifically, transitive probabilities, McDonald and Shillcock, 2003a,b) may be a specific instance of contextual constraint that captures the likelihood of words being used together in particular linguistic contexts (Frisson et al., 2005). The findings that a processing advantage for collocations is observed in contexts where the phrase is predicted or not provide some evidence that this processing advantage is not contextually driven.

The present findings add to evidence of the importance of multi-word sequences in reading. There is growing evidence that readers can exploit the redundancy in the linguistic input provided by various types of formulaic language, including the collocations investigated in the present experiments, but also idioms and spaced compounds (e.g., Siyanova-Chanturia et al., 2011; Cutter et al., 2014; Yu et al., 2016). Experiments like those presented here show that knowledge of such expressions can speed language processing, while other evidence suggests that the use of collocations reduces the burden on working memory (e.g., Millar, 2011). Some researchers have argued that the repeated use of a collocation or other formulaic expressions alters how linguistic information is represented in the mental lexicon (Croft and Cruse, 2004; see also Bybee, 2006). Specifically, some researchers propose that through repeated exposure formulaic language including collocations becomes lexicalized so that multi-word sequences are mentally-represented as a single lexical unit (e.g., Wray, 2002; Underwood et al., 2004; Conklin and Schmitt, 2008, 2012; Siyanova-Chanturia et al., 2011; Zang, 2019). The present findings contribute to debate about this issue by showing that the relative frequency of usage of “strong” and “weak” collocations can influence eye movements during reading, and that this effect cannot be explained simply in terms of a specific form of contextual constraint.

This and other evidence about the processing of formulaic language is also valuable to the advance of computational models of reading. As mentioned in the Introduction, the existing models, such as the E-Z Reader model (Reichle et al., 1998, 2003), base their fundamental assumptions on the computation of lexical frequency within words, rather than phrases. The present findings contribute to the increasing amount of evidence indicating that readers’ eye movements are influenced by the frequency of usage of formulaic sequences such as idioms, spaced compounds, and collocations. Such findings demonstrate that eye movement control in reading is sensitive to frequency effects for linguistic units that include more than one word. Accordingly, such findings suggest that the existing models of eye movement control during reading may require adjustments that consider the frequency of usage of multi-component units and individual words, in order to explain the complete linguistic frequency effects in reading.

## Data availability statement

The datasets presented in this study can be found in online repositories. The names of the repository/repositories and accession number(s) can be found in the article/supplementary material.

## Ethics statement

This experiment received ethical approval from the research ethics committee at Zhejiang University, and was conducted in accordance with the principles of the Declaration of Helsinki. The participants provided their written informed consent to participate in this study.

## Author contributions

HL, XW, and DL designed the experiments. HL collected and analysed the data. HL and KP wrote the manuscript. DL provided the data source. HZ revised the research materials. XW and DL gave critical comments. All authors contributed to the article and approved the submitted version.

## Funding

The research was supported by a Major Project of National Social Science Fund of China (14ZDB155), and a Humanities and Social Science Foundation grant from the Education Ministry of the People’s Republic of China (No. 19YJC740027).

## Conflict of interest

The authors declare that the research was conducted in the absence of any commercial or financial relationships that could be construed as a potential conflict of interest.

## Publisher’s note

All claims expressed in this article are solely those of the authors and do not necessarily represent those of their affiliated organizations, or those of the publisher, the editors and the reviewers. Any product that may be evaluated in this article, or claim that may be made by its manufacturer, is not guaranteed or endorsed by the publisher.

## Footnotes

^1^Following Vilkaite (2016), we conducted additional analyses that included mutual information and phrasal frequency as continuous variables, using the formula lmer10 = lmer(depvar ~context*type_coll+MI + phrasal_frequency + (1|pp) + (1|stim), data = data). For the collocation phrase, this produced a main effect of collocation type in first-pass reading times (*β* = 50.09, *SE* = 23.68, *t* = 2.15, *p* = 0.032), and main effects of collocation type and context in regression-path reading times (collocation type, *β* = 15.78, *SE* = 39.52, *t* = 3.99, *p* < 0.001; context, *β* = 53.71, *SE* = 23.40, *t* = 3.30, *p* = 0.025), with no indication of an interaction. No effects of mutual information or phrasal frequency were observed, suggesting that while these variables were likely to be important, their contribution was accounted for by the manipulation of collocation status, which was a better predictor in the model. For the collocation noun, the analysis produced main effects of collocation type and context in regression-path reading times (collocation type, *β* = 13.07, *SE* = 39.39, *t* = 3.32, *p* < 0.001; context, *β* = 46.63, *SE* = 31.30, *t* = 2.19, *p* = 0.032) and a main effect of collocation type in total reading times (collocation type, *β* = 82.81, *SE* = 24.52, *t* = 3.38, *p* < 0.001), with no indication of an interaction. No effects of mutual information or phrasal frequency were observed, again suggesting that this contribution was accounted for by the manipulation of collocation status.

^2^As with Experiment 1, we conducted additional analyses that included mutual information and phrasal frequency in the models as continuous variables. For the collocation phrase, this produced main effects of collocation strength and context in first-pass reading times (collocation strength, *β* = 25.26, *SE* = 7.97, *t* = 3.17, *p* < 0.001; context, *β* = 39.99, *SE* = 9.43, *t* = 4.24, *p* < 0.001), and regression-path duration (collocation strength, *β* = 45.32, *SE* = 12.77, *t* = 3.55, *p* < 0.001; context, *β* = 71.66, *SE* = 16.72, *t* = 3.55, *p* < 0.001), with no indication of an interaction. These analyses produced significant effects of phrasal frequency but not mutual information. For the collocation noun, the analysis produced a main effect of collocation strength in gaze duration (*β* = 11.20, *SE* = 4.90, *t* = 2.23, *p* = 0.022), single-fixation duration (*β* = 8.65, *SE* = 4.22, *t* = 2.05, *p* = 0.044), regression-path duration (*β* = 23.21, *SE* = 9.02, *t* = 2.57, *p* = 0.010), and total reading times (*β* = 11.20, *SE* = 4.90, *t* = 2.23, *p* = 0.022), with no effects of context or an interaction. These analyses produced significant effects of phrasal frequency but not mutual information. The indication from both sets of analyses is that the dichotomized variable of collocation strength had predictive power even when mutual information and phrasal frequency were included in the model as continuous variables.
